# Tetra-O-methyl nordihydroguaiaretic acid (Terameprocol) inhibits the NF-κB-dependent transcription of TNF-α and MCP-1/CCL2 genes by preventing RelA from binding its cognate sites on DNA

**DOI:** 10.1186/1476-9255-7-59

**Published:** 2010-12-07

**Authors:** Akinbolade O Oyegunwa, Michael L Sikes, Jason R Wilson, Frank Scholle, Scott M Laster

**Affiliations:** 1 Department of Microbiology, North Carolina State University, Raleigh, North Carolina, 27695-7615, USA

## Abstract

**Background:**

Tetra-O-methyl nordihydroguaiaretic acid, also known as terameprocol (TMP), is a naturally occurring phenolic compound found in the resin of the creosote bush. We have shown previously that TMP will suppress production of certain inflammatory cytokines, chemokines and lipids from macrophages following stimulation with LPS or infection with H1N1 influenza virus. In this study our goal was to elucidate the mechanism underlying TMP-mediated suppression of cytokine and chemokine production. We focused our investigations on the response to LPS and the NF-κB protein RelA, a transcription factor whose activity is critical to LPS-responsiveness.

**Methods:**

Reporter assays were performed with HEK293 cells overexpressing either TLR-3, -4, or -8 and a plasmid containing the luciferase gene under control of an NF-κB response element. Cells were then treated with LPS, poly(I:C), or resiquimod, and/or TMP, and lysates measured for luciferase activity.

RAW 264.7 cells treated with LPS and/or TMP were used in ChIP and EMSA assays. For ChIP assays, chromatin was prepared and complexes precipitated with anti-NF-κB RelA Ab. Cross-links were reversed, DNA purified, and sequence abundance determined by Q-PCR. For EMSA assays, nuclear extracts were incubated with radiolabeled probes, analyzed by non-denaturing PAGE and visualized by autoradiography.

RAW 264.7 cells treated with LPS and/or TMP were also used in fluorescence microscopy and western blot experiments. Translocation experiments were performed using a primary Ab to NF-κB RelA and a fluorescein-conjugated secondary Ab. Western blots were performed using Abs to IκB-α and phospho-IκB-α. Bands were visualized by chemiluminescence.

****Results**:**

In reporter assays with TLR-3, -4, and -8 over-expressing cells, TMP caused strong inhibition of NF-κB-dependent transcription.

ChIP assays showed TMP caused virtually complete inhibition of RelA binding in vivo to promoters for the genes for TNF-α, MCP-1/CCL2, and RANTES/CCL5 although the LPS-dependent synthesis of IκB-α was not inhibited. EMSA assays did not reveal an effect of TMP on the binding of RelA to naked DNA templates in vitro.

TMP did not inhibit the nuclear translocation of NF-κB RelA nor the phosphorylation of IκB-α.

**Conclusion:**

TMP acts indirectly as an inhibitor of NF-κB-dependent transcription by preventing RelA from binding the promoters of certain key cytokine and chemokine genes.

## Background

The NF-κB proteins are sequence-specific transcription factors that play critical roles in the immune system. NF-κB proteins regulate the expression of cytokines, chemokines, growth factors, and inflammatory enzymes in response to activation of T-cell, B-cell, Toll/IL-1R, and TNF-α receptors [1,2]. The NF-κB family of proteins is characterized by the presence of a conserved 300 amino acid Rel Homology Domain (RHD) which controls dimerization, DNA binding, and association with the inhibitory IκB proteins [3]. The five members of the mammalian NF-κB family; RelA (p65), RelB, c-Rel, NF-κB1 (p50) and NF-κB2 (p52) are present in unstimulated cells as homo- or heterodimers bound to inhibitory IκB proteins. This association prevents NF-κB proteins from translocating to the nucleus, thereby maintaining an inactive state [4]. In response to inflammatory stimuli such as TNF-α, IL-1, or LPS, multiple signaling pathways are activated resulting in the phosphorylation of IκB-α [5,6]. Subsequent poly-ubiquitination and proteosomal degradation of IκB-α permits the translocation of NF-κB proteins into the nucleus where transcription is activated [7,8]. NF-κB dimers exhibit variable binding affinities for consensus κB binding sites. These proteins also differ in their ability to initiate transcription; RelA, RelB and c-Rel have been shown to have potent trans-activating domains, while NF-κB proteins that lack transactivating domains such as p50 and p52 have been to shown to mediate transcriptional repression [3]. Activated NF-κB proteins can be inhibited by newly synthesized IκB proteins which cause re-export back to the cytosol [9].

Extracts of the Creosote bush, *Larrea tridentata*, found in deserts of the Southwestern United States and Northern Mexico, have been used for centuries by indigenous peoples to treat inflammatory disorders. Many of the medicinal effects of *L. tridentata *have been ascribed to the polyphenolic compound nordihydroguaiaretic acid (NDGA) [10]. In addition, *L. tridentata *also contains polyphenolic compounds with modifications to the backbone structure of NDGA [11]. A number of these compounds have been examined for their antiviral activity. For example, an analysis of eight methylated forms of NDGA for their ability to inhibit HIV replication revealed that tetra-O-methyl NDGA, also known as terameprocol (TMP), displayed the highest level of activity. Mechanistic studies suggest that TMP mediates this effect by inhibiting HIV Tat-mediated transactivation [12]. TMP has also been shown to block the replication of herpes simplex virus *in vitro *and this effect has been attributed to the drug's ability to block the binding of the transcription factor Sp1 to viral DNA, which is required for virus replication [13].

Based on these reports, we have recently evaluated the efficacy of TMP as an anti-inflammatory agent. We reasoned that since inflammation is heavily dependent on *de novo *transcription, TMP might be a useful therapeutic compound. We found that TMP exerted a range of effects on various inflammatory cytokines, chemokines and lipid mediators both *in vivo *and *in vitro *following treatment with LPS or infection with H1N1 influenza A virus strain PR/8/34 [14]. TMP strongly inhibited the production of TNF-α, MCP-1/CCL2, G-CSF, and several prostaglandins, while modestly inhibiting the production of IL-6 and MIP-1α/CCL3. Since the NF-κB RelA protein has been reported to regulate the expression of several of these genes [15-18]; we have focused our current studies on how TMP modulates RelA activation and occupancy at its cognate DNA binding motifs. We report that TMP did not affect the cytoplasmic activation and nuclear localization of RelA in RAW 264.7 cells following treatment with LPS. However, reporter assays revealed strong inhibition of NF-κB-dependent transcription. Chromatin immunoprecipitation (ChIP) assays revealed that TMP abrogated the LPS-induced binding of RelA at the TNF-α, MCP-1/CCL2, and RANTES/CCL5 promoters despite its inability to block NF-κB association with electrophoretic mobility shift assay (EMSA) probes *in vitro*. We conclude, therefore, that TMP acts indirectly to inhibit the binding of RelA to the promoters of certain key pro-inflammatory cytokine and chemokine genes. Taken together our data suggest that TMP could be useful for the treatment of inflammatory disorders where NF-κB RelA-dependent transcription plays a pathogenic role.

## Methods

### Cells and media

RAW 264.7 cells were obtained from the American Type Culture Collection (Manassas, VA) and were cultured in Dulbecco's modification of minimal essential medium (DMEM) with 4 mM L-glutamine, 4.5 g/L glucose, and 1.5 g/L sodium bicarbonate with 10% FCS. Media and supplements were obtained from Sigma-Aldrich, St. Louis, MO and Cellgro, Manassas, VA. FCS was obtained from Atlanta Biologicals, Atlanta, GA and Cellgro. Constitutive TLR3(293/TLR3-YFP), TLR8(293/TLR8) (InvivoGen, San Diego, CA) and TLR4(293/TLR4-YFP/MD2) (a gift from D. Golenbock) expressing HEK293 cells were grown in DMEM supplemented with 10% FCS, 1% antibiotics, 20 μg/ml gentamicin at 37°C. Stable expression of TLRs was maintained with the addition of 10 μg/ml blasticidin for (293/TLR3) and (293/TLR8) cells, and 400 μg/ml of G418 (Geneticin) for (293/TLR4) cells.

### Chemicals and biological reagents

Unless otherwise indicated, reagents were purchased from Sigma-Aldrich. TMP was supplied by Erimos Pharmaceuticals, Raleigh, NC. DMSO was used as the solvent for TMP in all experiments. The maximum DMSO concentration was 0.1% in all assays. This concentration of DMSO was tested in all assays and did not affect the results. LPS from *Salmonella Minnesota *R595 was purchased from LIST Biological Laboratories, Inc. (Campbell, CA).

### Quantitative RT-PCR analysis

Total RNA was extracted using the RNAeasy kit (Qiagen, Valencia, CA) according to the manufacturer's specifications. Residual genomic DNA was eliminated using on-column DNase digestion with the RNase-free DNase set (Qiagen) and resulting extracts were resuspended in nuclease free water. Amount and purity of RNA was determined using a Nanodrop 1000 spectrophotometer (ThermoFisher Scientific, Waltham, MA). RNA (1 μg) was denatured and reverse transcription was performed with the Improm ll reverse transcription kit (Promega, Madison, WI) in a reaction mix containing random hexamers as primers (50 ng/μl) for 60 min at 42°C. The iQTM SYBR Green supermix kit (BioRad, Hercules, CA), was used for Real-time PCR analysis. cDNA was amplified using primers specific for murine GAPDH, TNF-α, MCP-1/CCL2, and RANTES/CCL5 genes. Primer combinations are GAPDH [antisense: 5' ATG TCA GAT CCA CAA CGG ATA GAT 3'; sense: 5' ACT CCC TCA AGA TTG TCA GCA AT 3']; TNF-α [antisense: 5' AGA AGA GGC ACT CCC CCA AAA 3'; sense: 5' CCG AAG TTC AGT AGA CAG AAG AGC G 3']; MCP-1/CCL2 [sense: 5' CAC TAT GCA GGT CTC TGT CAC G 3'; antisense: 5' GAT CTC ACT TGG TTC TGG TCC A 3']; RANTES/CCL5: [sense: 5' CCC CAT ATG GCT CGG ACA CCA 3'; antisense: 5' CTA GCT CAT CTC CAA ATA GTT GAT 3']. All primer pairs were purchased from Integrated DNA Technologies (Coralville, IA). PCR was performed in 96 well plates (Eppendorf AG, Hamburg, Germany). Samples were amplified for a total of 50 cycles, followed by a meltcurve analysis to ensure the specificity of reactions. To generate a standard curve, total RNA was isolated from the cells and 300-600 bp fragments of the gene of interest were amplified by RT-PCR using cognate primer sets. PCR fragments were gel purified, quantified, and the copy number was calculated. Serial tenfold dilutions were prepared for use as templates to generate standard curves. All samples were normalized to amplified murine GAPDH. GAPDH control was analyzed per plate of experimental gene to avoid plate-to-plate variation. Final RT-PCR data is expressed as the ratio of copy numbers of experimental gene per 10^3 ^or 10^4 ^copies of GAPDH for samples performed in duplicates.

### Western blot analysis

After treatments, cell monolayers were washed twice with cold phosphate buffered saline (PBS), solubilized in lysis buffer (50 mM Hepes, pH 7.4, 1 mM EGTA, 1 mM EDTA, 0.2 mM sodium orthovanadate, 1 mM phenylmethylsulfonyl fluoride, 0.2 mM leupeptin, 0.5% SDS) and collected by scraping. The protein concentration for each sample lysate was determined using the Pierce BCA system (Pierce, Rockford, IL). Equal protein samples (25 μg) were loaded on 12% Tris-Glycine gels and subjected to electrophoresis using the Novex Mini-Cell System (Invitrogen). Following transfer, and blocking, blots were probed with antibodies specific for the phosphorylated serine 32 residue of IκB-α and total IκB-α protein (Cell Signaling; Beverly, MA). Bands were visualized using the SuperSignal Chemiluminescent system (Pierce).

### Immunofluorescence

RAW 264.7 cells were seeded onto 8 well chamber slides and stimulated with 1 μg/ml of LPS or co-stimulated with 1 μg/ml of LPS and 25 μM TMP for various amounts of time. To visualize NF-κB subcellular localization at the end of each treatment period, cells were briefly washed with phosphate-buffered saline, fixed in 4% paraformaldehyde, permeabilized with 0.1% Triton X-100, and blocked (2% bovine serum albumin, 5% normal horse serum, and 10 mM glycine in phosphate-buffered saline). The cells were then incubated with a rabbit monoclonal anti-NF-κB (p65) antibody (Santa Cruz Biotechnologies, Santa Cruz, CA), followed by incubation with a goat anti-rabbit fluorescein isothiocyanate-conjugated secondary antibody (Southernbiotech, Birmingham, AL). Fluorescence was viewed using a Zeiss Axioskop 2 microscope (Zeiss AG, Oberkochen, DE). Images were captured using a spot camera (Diagnostic Instruments, Inc., Sterling Heights, MI).

### Cytokine Measurements

MCP-1/CCL2 and TNF-α ELISA kits were purchased from R&D Systems (Minneapolis, MN), Assay Designs (Ann Arbor, MI) or eBioscience (San Diego, CA). RAW 264.7 cells were stimulated with 1 μg/ml of LPS for 24 hrs and supernatants were collected for ELISA assays. In each case, sample values were interpolated from standard curves. Optical density was determined using a PolarStar microplate reader (BMG Labtechnologies, Durham, NC).

### Reporter Assays

Reporter assays were performed using a luciferase gene under the control of an NF-κB response element (NF-κB -Luc; Stratagene, Santa Clara, CA). Briefly, the plasmid contains 5 consecutive NF-κB binding motifs designed from a consensus sequence cloned into a PGL3 vector. 100 ng each of NF-κB-Luc and pCMV beta (β-Gal) (Clontech) and 300 ng of pcDNA6 (Invitrogen) were cotransfected into 293/TLR3, 293/TLR4-YFP/MD2 and 293/TLR8 cells using the TransIT-LT1 transfection reagent (Mirus, Madison, WI). pcDNA6 was used to keep the overall DNA concentration at a total of 500 ng which has proven itself suitable for reporter assay in this system. At 24 h post-transfection, cells were either treated for 4 hours with 20 μg/ml poly(I:C) (pIC; Calbiochem, Gibbstown, NJ), 1 μg/ml LPS or 1 μg/ml resiquimod (R-848; Axxora, San Diego, CA) alone, or co-treated with 25 μM TMP. Following treatment, cells were lysed in reporter lysis buffer (Promega, Madison, WI) containing 0.1% Triton X-100 and assayed for Luc and β-Gal activities using a Promega Luc assay system and an ONPG (*o*-nitrophenyl-β-D-galactopyranoside)-based β-Gal assay. β-Gal activity was used to normalize the Luc data for all experiments. All data are expressed as relative light units/mU of β-Gal activity.

### Chromatin immunoprecipitation (ChIP) assays

4.5 × 10^7 ^RAW 264.7 cells were stimulated with 1 μg/ml LPS or co-treated with 1 μg/ml LPS and 25 μM TMP for 4 hours and chromatin was isolated by methods previously described [19]. Briefly, after treatments, cells were harvested and nucleoprotein complexes were crosslinked with formaldehyde (1% final) with shaking for 10 min at room temperature, followed by incubation with glycine (125 mM final) for an additional 5 min. Cells were pelleted, washed and resuspended in 500 μl lysis buffer (10 mM Tris-HCl, pH7.5, 10 mM NaCl, 3 mM MgCl_2_, and 0.5% NP-40) supplemented with 1 mM PMSF and 1× Protease Inhibitor Cocktail (PIC, Roche). Nuclei were pelleted and resuspended in Micrococcal nuclease buffer (10 mM Tris-HCl, pH 7.5, 10 mM NaCl, 3 mM MgCl_2_, 1 mM CaCl_2_, 4% NP-40) supplemented with 1 mM PMSF and 1× PIC, and chromatin was sheared with the addition of 10 U MNase for 7 min at 37°C. Digestion was stopped with the addition of EDTA (10 mM final), and the resultant chromatin was stored at -80°C. Shearing was confirmed by electrophoresis and >80% of the DNA was in fragments <400 bp.

Using magnetic capture, Protein A and G-coupled Dynabeads (Invitrogen) (5 μl each/IP) were washed 2× (100 μl/IP) in RIPA buffer (10 mM Tris-HCl, pH 7.5, 150 mM NaCl, 1 mM EDTA, 0.5 mM EGTA, 1% Triton X-100, 0.1% SDS, 0.1% NaDeoxycholate and sheared salmon sperm DNA (0.5 mg/ml). Beads were conjugated with 1-5 μg antibody for 1 h at 4°C in RIPA buffer supplemented with 1 mM PMSF and 1× PIC. Conjugated antibody:bead complexes were washed 3× in RIPA buffer as described above, and protein-DNA complexes were immunoprecipitated for 2 h at 4°C with rotation in RIPA buffer (100 μl) supplemented with 1 mM PMSF, 1× PIC and chromatin (10^5 ^cell equivalents). Following IP, beads were successively washed 4× in RIPA buffer and 2× in TE 8.0, and protein-DNA complexes were eluted in 100 mM NaHCO_3 _by gentle vortexing for 15 min at room temp. Supernatants were recovered and crosslinks were reversed in NaCl (100 mM final) together with matched input samples by heating at 95°C for 15 min. Proteins were removed using Proteinase K (10 μg/ml final) for 1 h at 45°C and DNA was purified using Qiaquick nucleotide removal columns (Qiagen) according to the manufacturer's instructions.

### ChIP Q-PCR and data analysis

For realtime PCR, bound (3 μl) and input samples were amplified in a MyIQ thermal cycler (Bio-RAD) using 1× SensiMix *Plus *(Quantace, London, UK) and primers specific for the RelA binding sites at the TNF-α, MCP-1/CCL2 and RANTES/CCL5 promoters. TNF-α: (forward:5'-TCTCAAGCTGCTCTGCCTTC-3'; reverse:5'CACCAGGATTCTGTGGCAAT-3'). RANTES/CCL5:(Forward:5'-TGGAGGGCAGTTAGAGGCAGAG-3';reverse:5'-AGCCAGGGTAGCAGAGGAAGTG-3') and MCP-1/CCL2:(Forward:5'-ATTCTTCCCTCTTTCCCCCCCC-3';reverse:5'-TCCGCTGAGTAAGTGCAGAGCC-3') Cycling parameters for 20 μl reactions were 95°C 10 min, followed by 50 cycles of 95°C, 20 s; 60°C, 30 s; 72°C, 30 s, for all genes listed. Fold enrichment in the bound fractions relative to input was calculated as previously described [20], and the average enrichment for triplicate amplifications was reported.

#### Electrophoretic mobility shift assays (EMSA)

RAW 264.7 nuclear extracts and radioactive probes were prepared and EMSA reactions performed as previously described [21]. Sequences of wildtype and mutant oligonucleotide EMSA probes include: wildtype TNF-α κB3 sense (5'-AACAGGGGGCTTTCC-3') and antisense (5'-AGGAGGGAAAGCCCC-3'), and mutant TNF-α κB3 sense (5'-AACAGGGGGCTGAGCCTC-3') and antisense (5'-GAGGCTCAGCCCCCTGTT-3').

### Statistical Analysis

All graphs and statistical analyses were produced using Prism software (GraphPad Software Inc., La Jolla CA).

## Results

### TMP acts early to inhibit synthesis of TNF-α and MCP-1/CCL2 mRNAs

**Figure 1:**
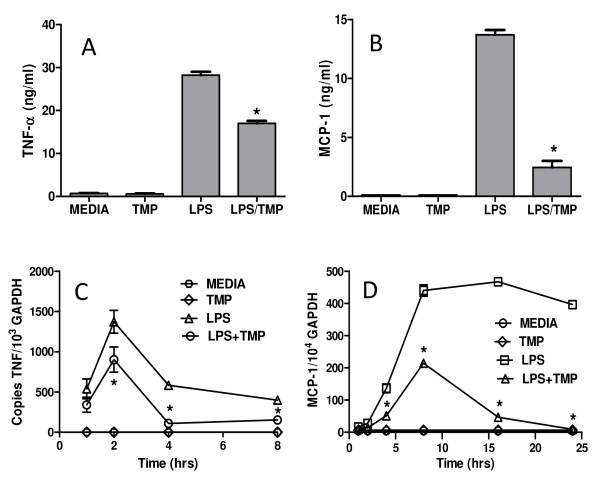
**TMP inhibits TNF-α and MCP-1/CCL-2 protein and mRNA**. RAW 264.7 cells were either stimulated with 1 μg/ml of LPS or 1 μg/ml of LPS and 25 μM TMP. Following 24 h of treatment, supernatants were collected and levels of TNF-α (A) and MCP-1/CCL2 (B) were determined by ELISA. To assess the effects of TMP on the transcription of TNF-α (C) and MCP-1/CCL2 (D) genes, RNA was prepared from RAW 264.7 cells stimulated with 1 μg/ml of LPS or 1 μg/ml of LPS and 25 μM TMP for the indicated time periods. Quantitative RT-PCR was used to analyze the levels of TNF-α and MCP-1/CCL2 mRNA. Asterisks indicate significant differences between treatments with LPS and LPS and TMP (p < 0.05, T-test).

We have previously shown that TMP inhibits the LPS-induced production of TNF-α and MCP-1/CCL2 from RAW 264.7 macrophage-like cells [14]. Representative experiments illustrating this effect are shown in Figures 1A and 1B. Typically, following a 24 h treatment with 1 μg/ml LPS in the presence of 25 μM TMP, levels of TNF-α and MCP-1/CCL2 are suppressed by 40 and 80%, respectively. Previously we found that the TMP-mediated reduction in these protein levels correlated with effects on accumulation of the specific mRNAs, leading us to speculate that TMP could interfere with transcription [14]. However, because regulation of cytokine and chemokine mRNA can be complex, we sought direct evidence for an early effect of TMP on mRNA synthesis. As shown in Figure 1C, the effect of TMP on the synthesis of TNF-α mRNA was evident early and maintained throughout the 8 h experiment [14] consistent with an effect on the transcriptional activation of the TNF-α gene. The rapid rise and fall in levels of TNF-α mRNA following treatment with LPS is typical and has been attributed to the action of various transcription factors [21,22] followed by tristetrapolin (TTP)-mediated mRNA degradation [23,24]. As shown in Figure 1D, an early effect of TMP on the synthesis of MCP-1/CCL2 mRNA was also noted; results that are again consistent with an effect of TMP on transcriptional activation. In this case, however, we also observed a reduction in steady state levels of MCP-1/CCL2 mRNA in the presence of TMP (Figure 1D). This effect was selective for MCP-1/CCL2 mRNA; TMP did not alter TNF-α mRNA expression kinetics.

### TMP inhibits NF-κB dependent reporter activity

**Figure 2:**
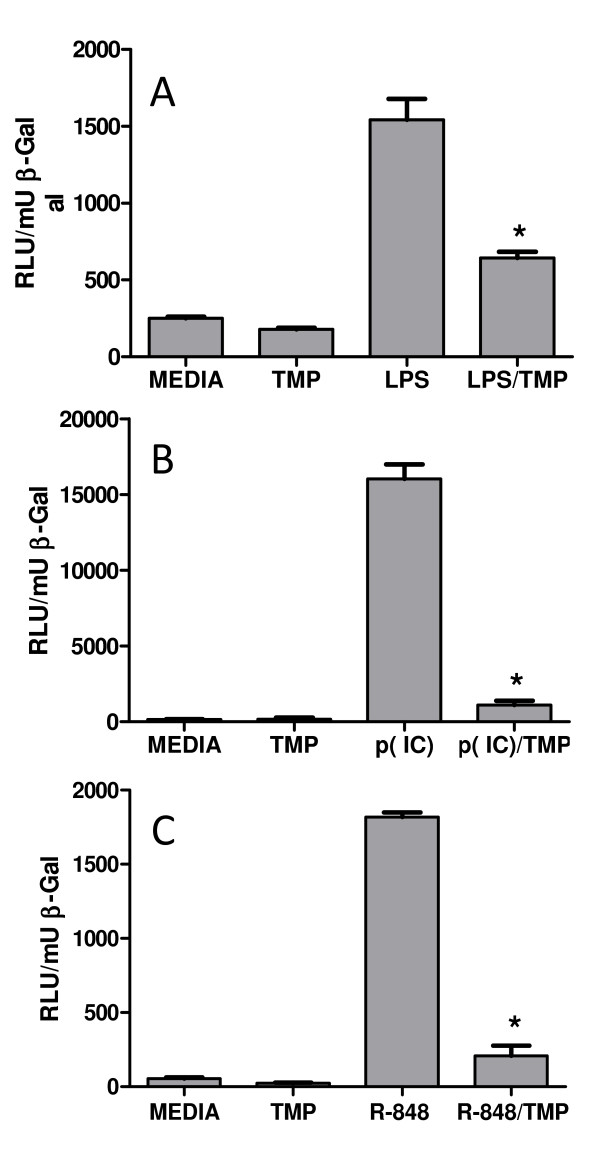
**TMP represses NF-κB dependent reporter activity**. The effect of TMP on LPS induced TLR4 signaling was evaluated by reporter analysis. HEK293/TLR4-YFP/MD2 cells were co-transfected with NF-κB -Luc and β-gal control plasmids then, after 4 hours of treatment, luciferase activity was measured in cell lysates (A). To analyze the effects of TMP on other TLR family members HEK293/TLR3 (B) and HEK293/TLR8 (C) cells were co-transfected as above, treated for four hours with 10 μg/ml poly(IC) (B) or 1 μg/ml R-848 (C) and/or 25 μM TMP, and luciferase activity determined in cell lysates. Each experiment was performed at least 3 times and representative experiments are shown. Asterisks indicate significant differences between ligand treatments and ligand treatments with TMP (p < 0.05, T-test).

NF-κB proteins, primarily RelA/NF-κB1 heterodimers, have been reported to play a key role in the transcriptional activation of cytokine genes after LPS stimulation [25]. Therefore, we hypothesized, that the inhibitory effects of TMP on the transcription of TNF-α and MCP-1/CCL2 mRNAs might stem from the effect of the drug on the activity of NF-κB proteins. To test this hypothesis, we performed reporter assays with cells expressing an NF-κB response element. HEK293 cells co-expressing TLR4 and MD2 (a co-receptor needed for TLR4 signaling) (HEK293/TLR4-YFP/MD2) were stimulated with 1 μg/ml of LPS or 1 μg/ml of LPS and 25 μM of TMP for a period of 4 hours and cell lysates were analyzed for NF-κB dependent luciferase activity. As shown in Figure 2A, LPS stimulation strongly increased NF-κB dependent reporter activity approximately 7 fold. This effect was inhibited by TMP by approximately 60%, a result consistent with the hypothesis that TMP inhibits the activity of NF-κB. This effect was dose dependent with a concentration of 12.5 μM TMP inhibiting NF-κB reporter activity by 35% (data not shown). It should also be noted that western blots with a TLR-4 specific Ab did not reveal an effect of TMP on the expression of TLR-4 following transfection (data not shown).

The possibility that TMP was affecting the activity of LPS and/or its receptor, as opposed to NF-κB-dependent transcription, was examined by testing the effects of TMP on TLR-3 and TLR-8-mediated activation of NF-κB [26,27]. The natural ligands for TLR-3 and TLR-8 are double and single stranded RNA, respectively. In these experiments we used the artificial ligands poly(I:C) for TLR-3 and resiquimod (R-848) for TLR-8. HEK293/TLR3 and HEK293/TLR8 cells were stimulated with either 20 μg/ml poly(I:C) or 1 μg/ml R-848, respectively. As with LPS, we found that TMP blocked both poly(I:C)- and R-848-induced, NF-κB-dependent reporter activity (Figures 2B and 2C, respectively). Taken together these data suggest that TMP mediates a broad, receptor-independent, inhibitory effect on NF-κB-dependent transcription.

### TMP inhibits RelA binding to its cognate motifs in vivo

**Figure 3:**
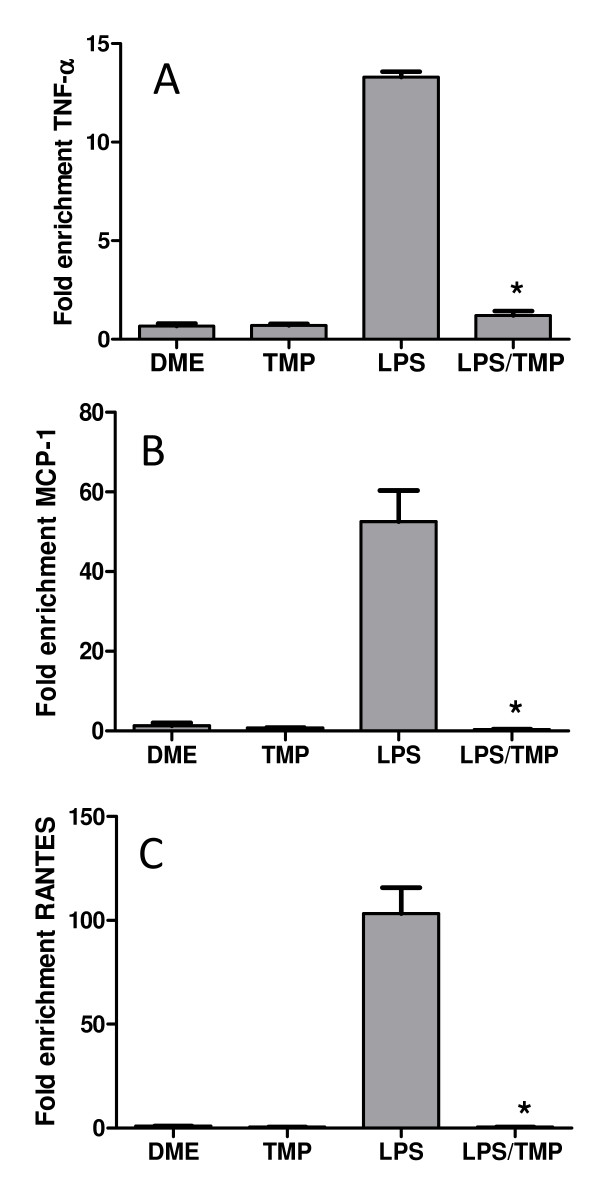
**TMP inhibits RelA DNA binding**. RAW 264.7 cells were either stimulated with 1 μg/ml of LPS or 1 μg/ml of LPS and 25 μM TMP for 4 hours. Following treatment, protein:DNA complexes were cross-linked, and RelA binding at the TNF-α (A), MCP-1/CCL2 (B) and RANTES/CCL5 (C) promoters was assessed by chromatin immunoprecipitation. Enrichment was calculated relative to pre-IP input control levels and was normalized to signals obtained with non-specific IgG control antibodies. Data shown are representative of two independent experiments and chromatin preparations. Asterisks indicate significant differences between LPS treatments and LPS treatments with TMP (p < 0.05, T-test).

ChIP assays were used next to confirm this hypothesis and to gain insight into the mechanism of NF-κB inhibition. Furthermore, with these assays we could examine RelA activity specifically since this is the major NF-κB protein responsible for cytokine and chemokine transcription following LPS stimulation [5]. RAW 264.7 cells were treated with LPS and/or TMP, the resulting nucleo-protein complexes were cross-linked, and RelA specific antibodies were used to precipitate RelA:DNA complexes. DNA was subsequently purified and analyzed by quantitative RT-PCR using primers specific for the NF-κB binding sites on the TNF-α, MCP-1/CCL2, and RANTES/CCL5 promoters. RANTES/CCL5 was included since it's promoter does contain NF-κB binding sites, although previous studies showed that its expression was not blocked by TMP. As shown in Figure 3, treatment with LPS strongly enhanced the binding of RelA to each promoter, an effect that was completely blocked by treatment with TMP. We conclude, therefore, that TMP prevents NF-κB-dependent transcription by preventing RelA from binding to its cognate motifs on the DNA in vivo.

### TMP does not directly inhibit RelA:DNA binding

**Figure 4:**
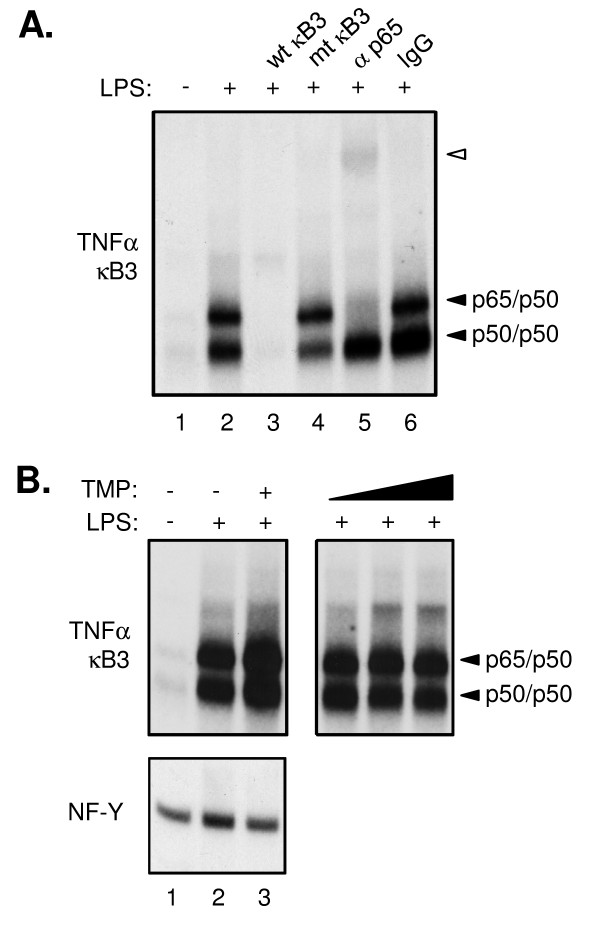
**TMP does not impair NFκB binding *in vitro *to the TNF-α promoter**. (A) Nuclear extracts from untreated (*lane 1*) RAW 264.7 cells or cells stimulated 4 hrs with 1 μg/ml LPS (*lanes 2-6*) were incubated with a radiolabeled ds oligonucleotide probe to the κB3 site of the TNF-α promoter. Probes were incubated with nuclear extract alone (*lanes 1 and 2*), in the presence of 100-fold molar excess of unlabeled wt (*lane 3*) or mutant κB3 competitor oligonucleotides (*lane 4*), or in the presence of the indicated Abs (*lanes 5 and 6*). Specific nucleoprotein (*filled arrows*) and Ab-supershifted complexes (*empty arrows*) are indicated. (B) The impact of TMP on protein binding to TNF-α κB3 (upper panels) or control NF-Y (bottom panel) was assessed in nuclear extracts from LPS-treated RAW 264.7 cells co-stimulated with TMP (*lane 3*) or upon addition of exogenous TMP to the binding reaction (*lanes 4-6*, 0.25 μM, 2.5 μM, and 25 μM, respectively).

Loss of RelA binding at the TNF-α promoter in our ChIP analyses suggests that TMP either directly inhibits RelA:DNA binding or acts indirectly to alter assembly of the TNF-α promoter nucleoprotein complex. To determine if TMP competitively impairs RelA:DNA binding, we tested the ability of NF-κB nuclear proteins to bind radiolabeled ds oligonucleotides of cognate κB sites on the TNF-α promoter by EMSA (Figure 4). LPS treatment of RAW 264.7 cells induced high levels of nuclear protein binding to a radiolabeled probe of the κB3 site (-311 relative to the TNF-α transcription start site) (Figure 4A, compare lanes 1 and 2). Binding was readily competed by unlabeled wildtype κB3 probe (Figure 4A, lane 3), whereas a 3-base substitution in the probe abolished competition (lane 4). The ability of anti-p65 antibody to specifically supershift the upper nucleoprotein complex (lane 5) confirms the identity of this band and recapitulates recent findings in LPS-treated RAW 264.7 cells [28]. In contrast to our *in vivo *ChIP analyses, addition of 25 μM TMP during LPS induction of RAW 264.7 cultures had no apparent impact on NF-κB binding at either the κB3 (Figure 4B, lane 3) or κB2 sites of TNF-α (data not shown). Likewise, NFκB binding was unaffected when nuclear extracts from LPS-treated RAW 264.7 cells were pre-incubated with varying concentrations of TMP prior to addition of the radiolabeled DNA probe (Figure 4B, lanes 4-6), suggesting that TMP does not directly inhibit NFκB binding to DNA.

### TMP does not inhibit the nuclear translocation of NF-κB RelA

**Figure 5:**
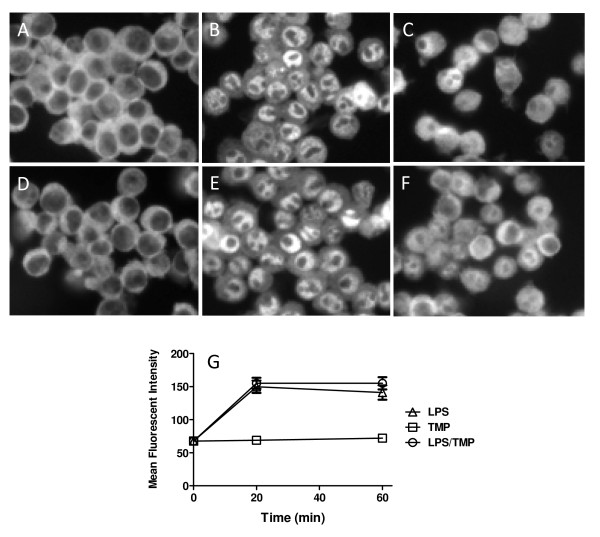
**TMP does not prevent nuclear translocation of NF-κB**. Cells were either left untreated (A) or treated with LPS (1 μg/ml) for 20 (B) or 60 min (C) then stained. In panel D, cells were treated with 25 μM TMP for 60 min while panels D and F show treatments with LPS and TMP for 20 and 60 min, respectively. Following treatment cells were fixed, permeabilized and stained with anti-RelA Ab and a fluorescein coupled secondary Ab. Representative images from a single experiment are shown in A-F. For G, Photoshop (Adobe) was used to analyze images and determine mean fluorescence intensity for the nuclear region of 120 cells at each time point for each variable (20 cells from two fields from three independent experiments). Treatment with LPS and LPS in combination with TMP did not produce significant differences (p < 0.05, T-test).

Antibody to RelA was used in immunofluorescence experiments to determine whether TMP blocked the nuclear translocation of RelA. As shown in Figures 5A-C, LPS treatment of RAW 264.7 cells caused strong nuclear translocation of RelA; twenty min. after treatment with LPS was initiated virtually all cells displayed nuclear RelA (Figure 5B). At later time points nuclear staining became more diffuse but overall staining intensity in the nuclear region of the cells remained relatively constant (Figure 5C and 5G). As shown in Figures 5D-G, TMP did not affect this process. Nuclear staining was evident in virtually all cells 20 min. after treatment with LPS was initiated and signals remained high at subsequent time points. TMP also failed to affect the translocation of RelA in C3HA mouse fibroblasts and NTERA-2 neuronal cells following treatment with LPS (data not shown). Together, these results suggest that TMP does not interfere with signaling to, and movement of RelA into the nucleus following treatment with LPS.

### TMP does not affect the phosphorylation of IκB-α

**Figure 6:**
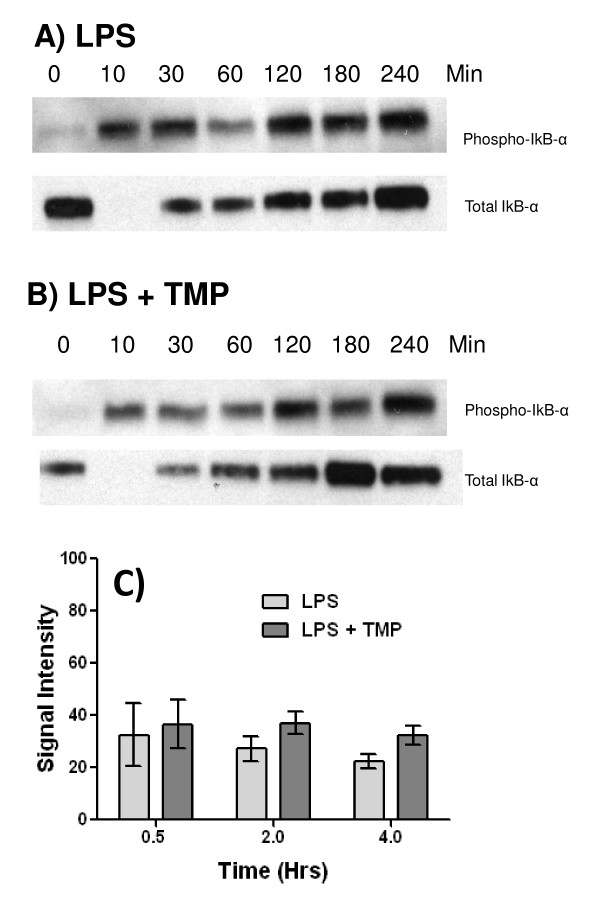
**TMP fails to affect IκB-α phosphorylation**. RAW 264.7 cells were stimulated with either 1µg/ml of LPS (A) or 1µg/ml of LPS and 25µM TMP (B) for the indicated times. Lysates were prepared and analyzed by western blot with Abs specific for the phosphorylated serine 32 residue of IκB-α and total IκB-α. Representative experiments are shown in panels A and B. For densitometric analysis (C), phospho-IκB-α blots were scanned and band intensity determined using Photoshop. Values shown are means +/- SEM from three independent experiments. Treatment with LPS and LPS in combination with TMP did not produce significant differences (p < 0.05, T-test).

Finally, to confirm this hypothesis we examined the effects of TMP on the LPS-induced phosphorylation of IκB-α, the final step in the signaling cascade, which results in dissociation of the RelA/p50 heterodimer from IκB-α, permitting nuclear translocation of RelA/p50 [29]. As shown in Figure 6A, we found that LPS stimulation induced phosphorylation of IκB-α within 10 mins and that levels of phospho-IκB-α remained relatively constant for up to 4 hours. Note that levels of total IκB-α drop below levels of detection at the 10 min. time point (Figure 6A). According to the antibody manufacturer, this occurs because phosphorylation of IκB-α is complete and this modification blocks the binding of the total IκB-α antibody. Detection of total IκB-α at later time points represents newly synthesized, non-phosphorylated molecules. As shown in Figure 6B, the pattern of IκB-α phosphorylation did not change in the presence of TMP. Small changes were noted from experiment to experiment however none of these effects were significant (Figure 6C). We conclude, therefore, that TMP is not interfering with signaling pathways that result in the activation and translocation of NF-κB. It should also be noted that TMP did not affect the resynthesis of total IκB-α, which is dependent on RelA ([30]), indicating that TMP does not inhibit the RelA dependent transcription of IκB-α.

## Discussion

Previously we showed that TMP could inhibit the expression of a number of cytokines and chemokines following stimulation with LPS [14]. The production of TNF-α, MCP-1/CCL2, and G-CSF were most strongly inhibited and we hypothesized that these effects might stem from effects on NF-κB RelA, which is thought to play a key role in the activation of these genes. The results of reporter and ChIP assays confirmed this hypothesis. We found strong inhibition of NF-κB-dependent transcriptional activation and loading of RelA to the promoters of several genes. Based on these results, a series of experiments was performed in an attempt to understand the molecular mechanism underlying this activity of TMP.

One hypothesis we considered was a direct inhibitory effect of TMP on the interaction between RelA and its cognate sites on the DNA. TMP could be acting on RelA itself, binding to conserved motifs present in the amino terminus RHD thereby preventing RelA from recognizing its DNA binding site. Alternatively, TMP could be interacting with the DNA, preventing RelA from occupying its binding sites. In support of this hypothesis, Chen et al., [13] have shown that TMP can bind the HSV ICP4 promoter and prevent Sp1 binding. Additionally, compounds with structures similar to TMP; 3'-*O*-methyl NDGA [13,31] and tetra-O-glycyl-NDGA [32] have been shown to bind DNA and prevent Sp1 binding. The recent finding that Sp1 can directly bind to certain NF-κB sites on the DNA [33] also supported this hypothesis and raised the possibility that it is the same activity of TMP that is responsible for both RelA and Sp1 inhibition of binding. However, the results of our EMSA experiments did not support this hypothesis. TMP did not interfere with the abilty of RelA to bind its cognate site when TMP was incubated with cells prior to nuclear extract preparation. Similarly, TMP did not inhibit RelA:DNA binding when it was added *in vitro *to the nuclear extracts and DNA. We conclude, therefore, TMP is working indirectly, upstream of DNA binding in the NF-κB pathway to prevent RelA from loading its promoter following LPS stimulation.

We next considered the hypothesis that TMP inhibits the signaling pathway that results in RelA translocation into the nucleus. TLR3/8 transcription was blocked more effectively than was TLR4. Since both TLR3 and 8 are localized to endosomal compartment this difference could suggest an effect of TMP on endocytosis. However, the phosphorylation of IκB-α and nuclear translocation of RelA were not altered following treatment with TMP suggesting that TMP is affecting additional regulatory systems. The results of our experiments also showed that, in the presence of TMP, IκB-α was resynthesized normally after treatment with LPS. Transcription of IκB-α is dependent on RelA [30] suggesting that the effect of TMP is selective for only certain RelA:promoter interactions. Phosphorylation of RelA is a mechanism that has been shown to confer selectivity for certain promoters. For example, phosphorylation at Ser^276 ^has been shown to be critical for transcription of IL-8 and GROβ/CXCL2 but not IκB-α [34]. RelA which is phosphorylated at this site interacts with positive transcription elongation factor b (PTEF-b), which is required for IL-8 and GROβ/CXCL2 transcription but not IκB-α [34]. Similarly, phosphorylation at Ser^311 ^has been shown to regulate the interaction of RelA with other transcriptional coactivators such as cyclic AMP-responsive element binding protein/p300 and RNA polymerase II [35-37] while acetylation of RelA is also known to be a molecular switch that regulates its activity [38]. Clearly future experiments with TMP will need to evaluate its effects on the post-translational modification of RelA.

The range of inhibitory effects seen with TMP with different cytokines and chemokines may arise from the differential requirements of these genes for the various modified forms of RelA as discussed above. Alternatively, the variation might stem from the degree to which NF-κB RelA is required for transcription of each gene. For example, several groups have reported that transcriptional activation of the TNF-α and MCP-1/CCL2 genes is strongly dependent on the trans-activating activities of NF-κB RelA [17,39], likely explaining the strong inhibition of these molecules by TMP. Similarly, inhibition of NF-κB RelA binding might explain the strong inhibition of G-CSF production by TMP we noted previously [14]. NF-κB binding sites have been shown to be present at the G-CSF promoter (CSF box) [40] and nuclear factors have been shown to associate with these sequences. In contrast, TMP only weakly inhibited production of IL-6, MIP-1α/CCL3, and RANTES/CCL5 [14]. It is possible that for these genes, although NF-κB sites are present in their promoters, their transcription in RAW 264.7 cells treated with LPS is not predominantly dependent on NF-κB. Transcription of IL-6, for example, can be entirely dependent on NF-IL-6 (C/EBPβ) [41]. Similarly, while the MIP-1α/CCL3 LPS response element does contain an NF-κB c-rel binding site it also contains four C/EBP family binding sites [42]. For RANTES/CCL5, although Fessele, et. al. [43] reported that NF-κB is essential for LPS-induced transcription in mono mac 6 cells [43] Shin et. al. [44] observed that NF-κB is not required for its LPS-induced transcription in RAW 264.7 cells [45] (the cells we used in our investigation). In agreement, our ChIP assays showed complete inhibition of RelA binding to the RANTES/CCL5 promoter, while at the same time levels of RANTES/CCL5 mRNA and protein were not blocked by TMP [14].

In addition to the effects we noted on NF-κB, in our experiments we also noted an effect of TMP on the steady state levels of MCP-1/CCL2 mRNA (Figure 1D). To our knowledge, post-transcriptional regulation of MCP-1/CCL2 mRNA has not been reported. It is possible, that the effects of TMP may be related to the normal regulation of this mRNA. If levels of TTP-mediated degradation are normally low, they may be masked by the high levels of LPS-induced MCP-1/CCL2 transcription and only revealed when transcription is effectively blocked by TMP. In support of this hypothesis, MCP-1/CCL2 mRNA does contain the TTP AUUUA recognition site in its 3' untranslated region. It is also possible that TMP could be modifying TTP or the 3' untranslated region to enhance rates of degradation. If so, then one might also predict enhanced rates of TNF-α message degradation, which did not occur.

In summary, we have examined the effects of TMP on NF-κB activation, translocation and binding. We report that TMP inhibited NF-κB- dependent transcription and NF-κB RelA binding at the promoters of TNF-α, MCP-1/CCL2, and RANTES/CCL5. Since NF-κB RelA-dependent transcription is critical to numerous inflammatory and pathological responses, TMP might be useful to treat a variety of disorders. The safety of TMP has been established in several clinical trials, and testing for efficacy in inflammation should begin immediately.

## Conclusions

• TMP exerted an early inhibitory effect on the production of TNF-α and MCP-1/CCL2 mRNA from RAW 264.7 cells following treatment with LPS.

• TMP also accelerated the loss of MCP-1/CCL2 mRNA from RAW 264.7 cells following treatment with LPS.

• Reporter experiments with HEK293 cells showed that TMP can inhibit TLR3, TLR4, and TLR-8-dependent activation of NF-κB.

• ChIP assays showed that TMP can prevent the NF-κB RelA protein from binding its cognate sites on the DNA.

• Immunofluorescence experiments failed to reveal an effect of TMP on the nuclear translocation of RelA.

• Western blots failed to reveal an effect of TMP on the phosphorylation of IκB-α.

• EMSA assays failed to reveal an effect of TMP on the direct interaction between RelA and DNA.

• TMP should be considered as a candidate drug for the treatment of inflammation and pathology mediated by NF-κB.

## Abbreviations

TMP: terameprocol; LPS: lipopolysaccharide; TNF-α: tumor necrosis factor-α; MCP-1: monocyte chemotactic protein-1; NDGA: nordihydroguaiaretic acid; NF-κB: nuclear factor κB; q-PCR: quantitative reverse transcriptase polymerase chain reaction; TLR: toll like receptor; ChIP: chromatin immunoprecipitation; TTP: tristetrapolin; EMSA:electrophoretic mobility shift assay.

## Competing interests

Erimos Pharmaceuticals produced TMP but is no longer in existence. None of the authors was paid by Erimos nor did they have stock or shares in the company.

## Authors' contributions

AOO performed the experiments and drafted the manuscript. FS and JRW supervised the reporter assays. MLS supervised the ChIP and EMSA experiments. SML, FS, and MLS participated in design and coordination of the experiments, acquisition of funding, and drafting of the manuscript. All authors read and approved the final draft.
